# Reciprocal influence of per- and polyfluoroalkyl substances (PFAS) and soil organic matter on their fate in soils

**DOI:** 10.1007/s11356-025-37024-9

**Published:** 2025-10-10

**Authors:** Sajjad Hazrati, Jurate Kumpiene, Tiina Leiviskä, Ivan Carabante

**Affiliations:** 1 Waste Science and Technology, Luleå University of Technology, Luleå, Sweden; 2 Chemical Process Engineering, University of Oulu, Oulu, Finland

**Keywords:** PFAS, Soil organic matter, Dissolved organic matter, Hydrophobicity, PFAS leaching

## Abstract

The global accumulation of per- and polyfluoroalkyl substances (PFAS) in soils raises concerns about soil quality. While PFAS sorption may depend on the quality of soil organic matter (SOM), their unique properties may also affect SOM dynamics in complex and poorly understood ways, impacting long-term soil quality. Literature provides vague conclusions about how SOM, particularly its quality, influences PFAS–soil interactions and whether PFAS can modify SOM characteristics. The present study aims to enhance both the qualitative and quantitative understanding of the reciprocal impact that PFAS and SOM have on each other’s environmental fate. Sorption of three PFAS molecules and simultaneous mobilization of dissolved organic matter (DOM) in three distinct soils were studied. PFOS had the highest sorption by ranging 61–98% followed by PFOA and PFBA. ^13^C NMR analysis indicated that PFAS sorption is driven by hydrophobic components of SOM. The highest PFAS sorption was observed in soils containing polycyclic aromatic hydrocarbons (PAHs), while the lowest was recorded in soils with less hydrophobic SOM. Conversely, the presence of PFAS increased the release of DOM in soils with less hydrophobic SOM. The changes in DOM release induced by PFAS were directly influenced by the chemical properties of the soil components. Additionally, ^1^H NMR revealed notable structural changes in the chemical composition of DOM caused by PFAS, characterized by an increase in hydrophobic constituents and a decrease in hydrophilic components. The results indicated that PFAS can affect both the quantity and quality of SOM, potentially compromising long-term SOM stability and carbon sequestration in contaminated soils.

**Graphical Abstract:**

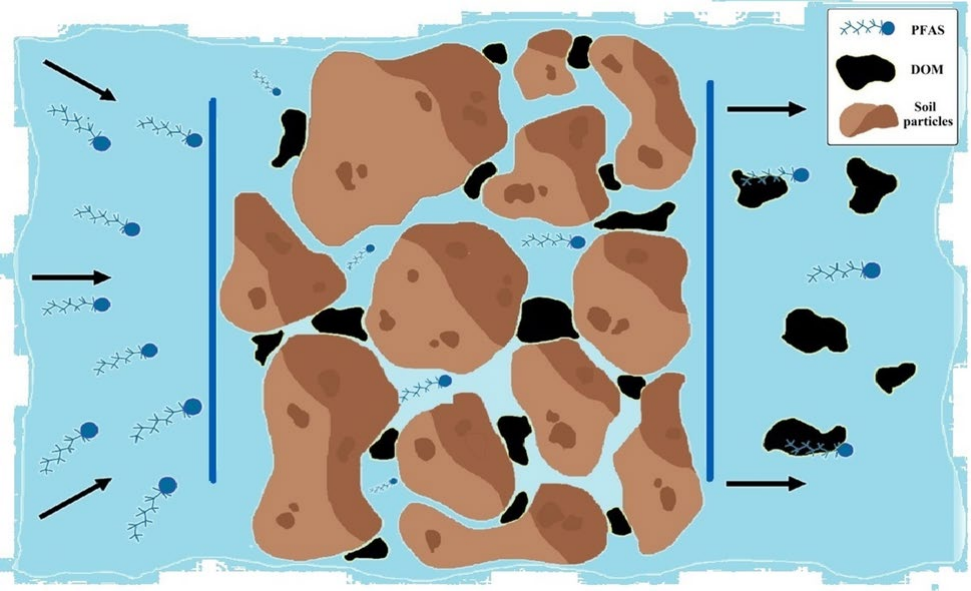

**Supplementary Information:**

The online version contains supplementary material available at 10.1007/s11356-025-37024-9.

## Introduction

Per- and polyfluoroalkyl substances (PFAS), characterized by their unique chemical attributes such as surface-active properties and persistent nature, are extensively employed in various industrial and consumer products (Gagliano et al. 2020; Martin et al. 2019). These chemical characteristics facilitate their widespread distribution and transport in soil and water, leading to their increasing accumulation in the soil matrix (Ambaye et al. 2022; Tansel et al. 2024). Numerous reports have documented the widespread presence of PFAS in the environment (Chokwe et al. 2024; Cui et al. 2020; Nannaware et al. 2024; Qiao et al. 2024). Also, several studies highlight the excessive accumulation of PFAS at contaminated sites, driven by both primary and secondary sources. For instance, high concentrations of PFOS have been reported at various sites, including 8250 µg kg^−1^ in Sweden (Filipovic et al. 2015), 13,400 µg kg^−1^ in Australia (Bräunig et al. 2019), and 36,534 µg kg^−1^ in the USA (McGuire et al. 2014), primarily linked to the use of firefighting foam in training areas. Other studies have identified PFAS pollution originating from application of municipal and industrial-derived biosolids on land, with reported concentrations of 878 µg kg^−1^ in USA (Brusseau et al. 2020) and 1692 µg kg^−1^ in Australia (Bräunig et al. 2017). The persistent nature of these chemicals enables them to resist biodegradation and environmental factors, earning them the label “forever chemicals” and allowing their accumulation over time (Bayode et al. 2024).

Due to their surfactant behavior and the presence of both hydrophobic and hydrophilic moieties within a single molecular structure, PFAS could be immobilized in soils either by electrostatic interaction, or by hydrophobic interactions (Alam et al. 2024; Wang et al. 2023). Due to their dualistic chemical nature, PFAS can potentially interact with various components in soil. Among these components, soil organic matter (SOM) is known as playing a vital role in the fate of PFAS in the environment (Dhulia et al. 2024; Sørmo et al. 2024). While SOM has been identified as a primary sink of PFAS in soils, the underlaying mechanisms of sorption remain poorly understood (Qi et al. 2022; Sörengård et al. 2020) and there is a lack of systematic, instrumental-based evidence on this topic. Some research has suggested that PFAS sorption on SOM is primarily linked to the organic carbon content in soil or sediment (Oliver et al. 2020). In contrast, other studies have highlighted the significance of the SOM chemistry on the PFAS adsorptive behavior (Campos-Pereira et al. 2022; Liu et al. 2019). Zhao et al. (2014), by applying ^13^C NMR spectroscopy, observed that the interaction between PFAS and OM significantly increased with higher aliphaticity in C components, accompanied by a decrease in aromatic and phenolic C constituents in the OM structure. Campos-Pereira et al. (2022) and Milinovic et al. (2015) emphasized that hydrophobic interactions were the dominant mechanism of interactions between PFAS and SOM.

The accumulation of PFAS in the soil matrix, particularly within the structure of SOM, has the potential to expose terrestrial carbon sources to risk. High levels of PFAS sorption in soils can potentially alter the chemical properties and reactivity of SOM both directly and indirectly (Xu et al. 2023). PFAS may compete with DOM for binding sites in the solid phase (Pannu et al. 2023), while in the liquid phase, their surfactant behavior enables them to act as both organic and inorganic ligands, enhancing the solubilization of organic matter (Wang et al. 2023). Additionally, PFAS can impact soil biodiversity and bacteria, potentially influencing the biodegradability and mineralizability of DOM in the soil. This, in turn, may lead to modifications to the carbon cycle and soil fertility (Cao et al. 2022; Qiao et al. 2018).

The mechanisms governing PFAS adsorption onto soil organic matter (SOM) remain poorly understood, and the effects of PFAS on SOM mobilization and chemical composition are still underexplored. This knowledge gap limits our ability to predict the environmental fate of PFAS. To address these questions, we investigated how SOM composition influences the sorption behavior of three PFAS compounds (PFOS, PFOA, PFBA) and examined whether PFAS exposure alters both the quantity and chemical characteristics of dissolved organic matter (DOM). This study focused on three soils with diverse SOM compositions and distinct physicochemical properties. The aim of the study was to (ⅰ) assess the sorption potential of PFAS in different soils and evaluate possible adsorption mechanisms based on the chemical composition of their SOM, as well as to (ⅱ) quantify DOM release from the soil matrix and investigate the potential changes in DOM chemistry caused by the presence of PFAS in the system. To assess chemical structures of SOM and detect potential changes in DOM, ^13^C NMR and ^1^H NMR analyses were implemented.

## Materials and methods

### Soil characteristics

Three soil samples showing distinct organic matter nature were used in this study. The first soil was a commercial gardening soil composed of natural peat, hereafter abbreviated as PT. The second soil contained a mixture of sand and moderately composted sewage sludge, hereafter abbreviated as CM. The third soil was collected from a contaminated site in Sweden and contained high amount of natural peat and high concentrations of polycyclic aromatic hydrocarbons (PAHs), hereafter abbreviated as PH. The characteristics of the soil samples were measured, and the methods and results are presented in Table S1 in the Supporting Information.

The homogenized soils were stored at their original moisture level. Before each analysis, the necessary portion of samples was air-dried and passed through a 2-mm mesh sieve. For the PH soil, characterization and sorption experiments were conducted under field-moist conditions to minimize PAH loss from the soil. Soil organic carbon was determined using a solid-state TOC analyzer (TOC-V CSH Shimadzu, Tokyo, Japan). The background PFAS concentration in soil samples was examined at a commercial laboratory (Eurofins Water Testing lab in Sweden) by liquid chromatography coupled with tandem mass spectrometry (LC–MS/MS) (Table S2). The 16 USEPA priority PAHs were measured in PH soil according to method based on USEPA 8270D, USEPA 8082 A, CSN EN ISO 6468, and USEPA 8000D using gas chromatography coupled with tandem mass spectrometry (GC–MS/MS) in ALS Scandinavia AB Laboratory (Table S3). The elemental composition of the surface of soil samples was measured by X-ray photoelectron spectroscopy (XPS) for native soils and after PFAS addition and results are presented in Table S4. XPS analysis was conducted using a Thermo Fisher Scientific ESCALAB 250Xi with a monochromatic Al Kα source (1486.6 eV). Charge correction was applied by calibrating the binding energy of adventitious carbon to 284.8 eV. Figure S1 presents the F 1 s XPS spectra, revealing fluorine species in native soil and a potential shift in binding energy following external PFAS addition.

### Batch sorption/release experiments

Batch experiments were conducted to simultaneously assess PFAS sorption in soils and its impact on DOM release. Pure chemicals, i.e., perfluorooctane sulfonate (PFOS; 96% purity, Sigma-Aldrich), perfluorooctanoic acid (PFOA; 96% purity, Sigma-Aldrich), and perfluorobutanoic acid (PFBA; 98% purity, Sigma-Aldrich), were used for the experiments. Two levels of PFOS, PFOA and PFBA concentration, 0.1 mg/L and 1 mg/L, along with a blank containing no PFAS, were studied (Details available in Table S5) both as a mixture (PFAS3) and individually, to evaluate the behavior of each compound in isolation and in the presence of the other compounds. The concentrations of PFOS, PFOA, and PFBA in mixture sample are listed in Table S5. Five grams of dry weight (dw) soil was transferred into 250-mL polyethylene bottles and suspended in a total solution volume of 200 mL, resulting in a soil-to-liquid ratio of 1:40.

The needed amount of PFAS from stock solutions (prepared using Milli-Q water as solvent) was added to the suspensions and samples were placed in rotating shaker with 10 rpm for 24 h at room temperature, based on preliminary kinetic tests (Figure S2). The pH in PFAS-contained samples was not controlled and PFAS addition did not change significantly the pH after equilibrium (Figure S3). After 24 h, the samples were filtered through 0.45-μm nylon filter. The DOC concentration in each sample was measured directly after filtering using a TOC analyzer (Shimadzu, Tokyo, Japan) and a portion of the collected supernatant freeze-dried to evaluate the structure of DOM using ^1^H NMR analysis. The rest of the filtered samples were stored at 4 °C in an airtight, dark environment until PFAS analysis was performed. The release of 16 USEPA priority PAHs from PH soil during the leaching test was measured by ALS Scandinavia Laboratory using GC–MS/MS (see Table S3 and Fig. S6).

### PFAS analysis

For quantification of PFAS, the three isotopically labeled standards (^13^C_8_-PFOS, ^13^C_8_-PFOA, and ^13^C_3_-PFBA, purity > 99%) was obtained from Wellington Laboratories (Guelph, ON). A total of 300 μL of the supernatant was transferred to 500-μL Eppendorf tubes, along with 175 μL of methanol containing 5 mM ammonium acetate, followed by the addition of 25 μL of the Mass-labeled internal standards (Wellington Laboratories, Guelph, Canada). The prepared samples were properly vortexed before analysis. PFAS concentrations were determined by direct injection into ultra-high performance liquid chromatography coupled with triple quadrupole mass spectrometry (UPLC-MS/MS, Acquity Premier–Xevo TQ-XS, Waters, USA). Detailed UPLC operating conditions and adjustments are provided in Table S6. Data analysis was performed using MassLynx V4.2 (Waters Laboratory Informatics), and manual checks and confirmation of integration were conducted for each peak. Limit of quantification for the examined compounds are presented in Table S7 in the Supporting Information.

### Characterization of SOM and DOM

For air-dried soil samples, cross-polarization magic angle spinning nuclear magnetic resonance (^13^C CPMAS NMR) spectra were recorded on a Bruker Avance III spectrometer equipped with a 4-mm MAS probe (Bruker, Billerica, USA). The spectrum was the result of adding 8192 scans, and 80-kHz proton decoupling (spinal 64) was employed. A Gaussian window function with a line broadening of − 10 Hz and Gaussian broadening of 0.01 was used in the processing. The obtained ^13^C NMR spectra were divided into seven chemical regions to integrate NMR spectra into different compound classes and describe the association of the carbon chemistry with chemical shift regions, based on a previous model (Baldock et al. 2004). Based on this model, the peak within the region of alkyl C (0–50 ppm) corresponds to the C of lipids and waxes in the structure of SOM (Kölbl and Kögel-Knabner 2004), which exhibits strong hydrophobic characteristics due to the aliphatic long carbon chains in their chemical structure (Gao et al. 2017; Murshid and Wang 2017). The chemical shift range from 50 to 112 ppm covers three distinct regions and corresponds to O/N-alkyl carbons in natural organic matter (OM). The 50–60 ppm region is primarily attributed to nitrogen-substituted carbon atoms, commonly found in amino acids and amino sugars (Knicker 2011). The 60–93 ppm region represents O-alkyl carbons, while the 93–112 ppm region corresponds to di-O-alkyl carbons, typically present in polysaccharides. Additionally, a characteristic peak around 74 ppm is often associated with carbon atoms in glucose units, as found in carbohydrate structural components such as cellulose and hemicellulose (Danchenko et al. 2020; Kögel-Knabner 2002). The chemical shift in the range of 112 to 140 ppm corresponds to aromatic carbons, such as those found in benzene, phenyl groups, or polyaromatic hydrocarbons. Phenolic and other oxygen-substituted aromatic carbons typically appear in the range of 140 to 165 ppm. Carbonyl carbons in carboxylic acids, esters, and amides are generally observed in the range of 165 to 190 ppm (Preston et al. 2009).

To achieve maximum sensitivity and the ability to detect slight changes in DOM composition in aqueous phase (collected supernatant), ^1^H NMR was applied. Thirty milligrams of the freeze-dried DOM samples were re-solubilized in 550 µL of D_2_O and transferred to NMR tubes. ^1^H NMR spectra for DOM samples were recorded on a Bruker 600 MHz Avance III HD spectrometer equipped with a BBO cryoprobe and a SampleJet sample changer (Bruker, Billerica, USA). ^1^H NMR spectra were recorded with presaturation to remove the residual water signal. Sixty-four scans were added with a relaxation delay of 2 s. Spectra were processed with a 2-Hz line-broadening. ^1^H NMR spectra were integrated based on Mitchell et al. (2018) and divided into seven chemical structures to provide a detailed interpretation of changes in the chemical structure of DOM (“DOM release affected by PFAS” section). According to Mitchell et al. (2018), the region between 0.6 and 1.3 ppm corresponds to aliphatic polymethylene and methyl groups, region 1.3–2.9 ppm shows N- and O-substituted aliphatic H, region 2.9–4.1 ppm shows O-alkyl H, region 4.1–4.8 ppm presents alpha protons of peptides, region 4.8–5.2 ppm shows anomeric protons of carbohydrates, region 6.2–7.8 ppm shows aromatic/phenolic H, and the region between 7.8 and 8.4 ppm represents amide H in DOM-derived ^1^H NMR spectra. Due to the use of D_2_O in preparing DOM samples for ^1^H NMR analysis, region 4 is dominated by water protons and was excluded from further calculations and interpretations.

### Quality control

High-density polyethylene (HDPE) bottles and Milli-Q water were used for the experiments. To avoid potential interference of methanol in the adsorption and DOC measurements, PFAS stock solutions were prepared in Milli-Q water, and their concentrations were consistently analyzed before each batch experiment. To prevent overestimation of DOC concentrations caused by PFAS-containing solutions, each PFAS stock solution was separately analyzed (in the absence of soil) using a TOC analyzer, and the measured values were negligible and showed no meaningful contribution to DOC. Blank samples were run in parallel with the test samples to check for any cross-contamination of the target compounds. All the results for blanks (without soil) were below the detection limit. Control tests (with known PFAS concentration, and without soil) were conducted for each batch of experiments to verify the repeatability of data in PFAS and DOC analysis, and the calculated standard deviations for PFAS and DOC repeatability were less than 5% and 4%, respectively. The relative standard deviation of triplicate sample analyses was calculated to assess the reproducibility of the method. The relative standard deviation for PFAS and DOC was within the range of 5–15% and 0.5–11%, respectively. The average recovery of PFOS, PFOA, and PFBA was 99.7 ± 6%, 96.8 ± 10%, and 104 ± 9%, respectively.

## Results and discussion

### Soil characterization

The chemical characterization of the soils is shown in Table S1. The major difference was observed in the content of organic carbon (OC) between soils. PT contained 45.1% followed by PH 29.9% and CM with 6.6% of OC. Although the OC content in PT soil was much higher than the OC content in CM soil, significantly lower DOC concentrations were observed in PT, with values of 262 mg/kg as compared to 412 mg/kg in CM. As presented in Table S3, PH soil contained 16 USEPA priority PAHs, with a total concentration of 8740 ± 1324 mg/kg (dw soil). The results of HNO_3_-extractable cations, shown in Table S1, revealed higher concentrations of Al and Fe in CM and PH which could be attributed to a higher content of Al/Fe oxides in these soils, which might contribute to the sorption of PFAS in these soils (Oliver et al. 2019). The SOM characterization by ^13^C NMR is depicted in Table 1; the spectra are shown in Figure S4. The table presents the percentage of carbon associated to the different chemical shift regions as well as the basic carbon chemistry (lipid/carbohydrate) based on the molecular mixing model (Baldock et al. 2004). Although low signal intensities were detected in CM, which might be due to the low organic matter (OM) content (Table S1), differences can be observed between the SOM in the soils. Results from the molecular mixing model, as shown in Table 1 and Figure S5, indicated that PT had the highest proportion of carbohydrates, whereas PH had the highest proportion of lipids. The lipid-to-carbohydrate ratio in PT and CM was 0.7 and 0.75, respectively. In contrast, the ratio in PH was nearly three times higher, which could indicate a higher level of hydrophobicity compared to the other soils (Kögel-Knabner 2002).

**Table 1:** ^13^C NMR-derived soil organic carbon. The integration model was employed to describe the association of the carbon chemistry with chemical shift regions, based on the findings of (Baldock et al. 2004)

Integrated C domains	Chemical shift region (ppm)	Unit	PT	CM	PH
Alkyl chains	0–50	%	21.14	24.33	41.63
Methoxy and N-alkyl groups	50–60	%	4.65	6.57	5.9
O-Alkyl	60–94	%	40.35	33.86	17.95
di-O-Alkyl/anomeric	93–112	%	10.47	10.2	8.71
Aromatic	112–140	%	8.8	11.78	12.72
Phenolic	140–165	%	4.86	8.05	7.32
Carbonyl	165–190	%	3.69	5.17	5.75
Alkyl C/O-alkyl C*			0.53	0.55	1.56
Carbon chemistry based on molecular mixing model					
Lipid		%	24.95	22.56	34.24
Carbohydrate		%	35.36	29.91	16.41
Lipid/carbohydrate			0.7	0.75	2.08

*A higher value of this ratio indicates greater hydrophobicity

### PFAS sorption by soils

**Fig. 1:**
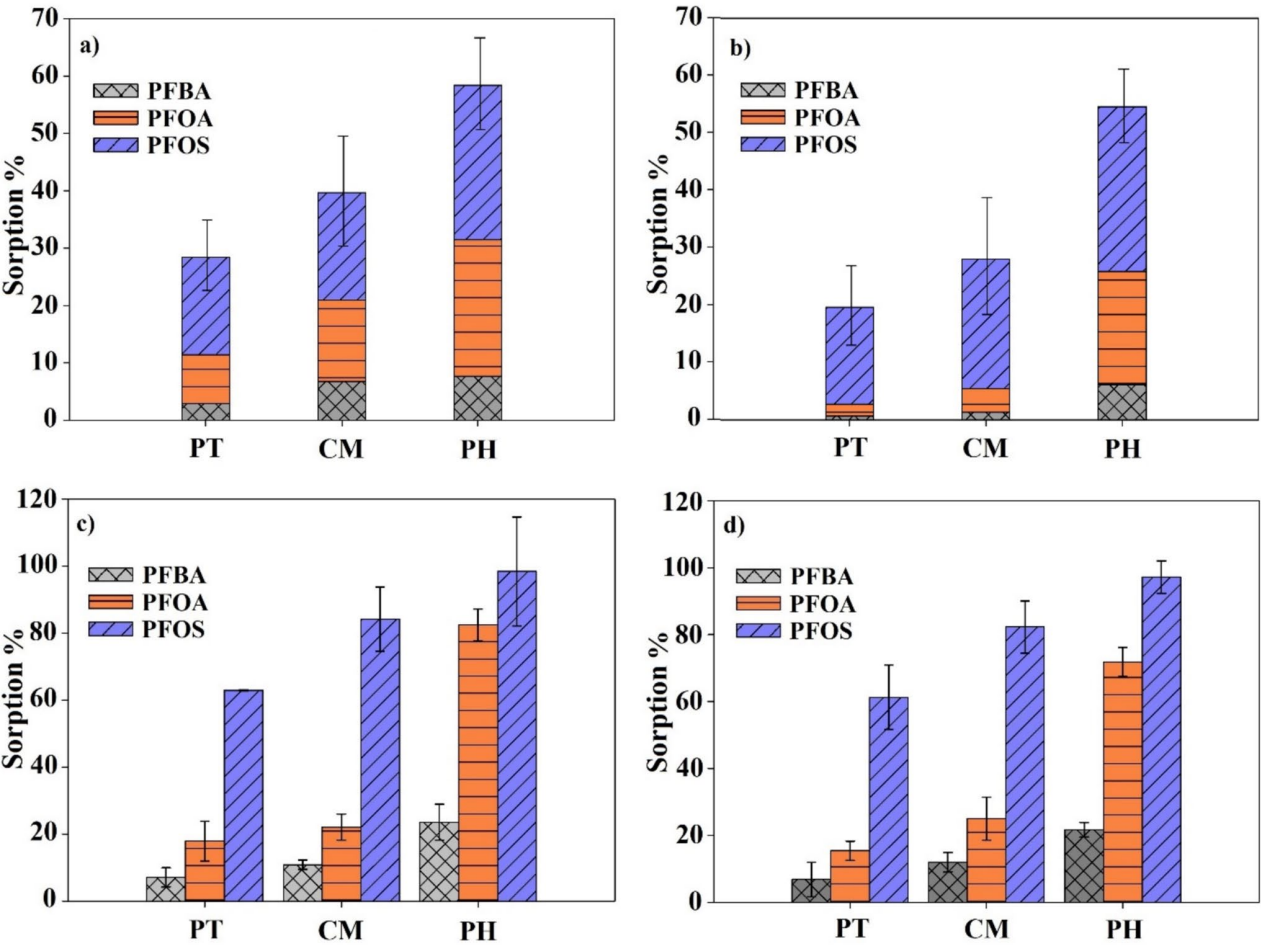
Sorption of PFOS, PFOA, and PFBA in soils both as a mixture and individual compound at 0.1 and 1 mg/L of PFAS concentration: sorption of (**a**) 0.1 mg/L of mixed PFAS, **b** 1 mg/L of mixed PFAS, **c** 0.1 mg/L of individual PFAS, and (**d**) 1 mg/L of individual PFAS. Error bars represent standard deviations (*n* = 3)

The sorption of two levels of PFAS mixture in soils is shown in Fig. 1a and b. The highest PFAS3 sorption was observed in PH soil followed by CM and PT soils. The sorption pattern was similar in both concentrations and PFOS exhibited the highest sorption potential in the mixture. This trend was also observed when individual PFAS compounds were examined (Fig. 1c, d). PFBA exhibited a lower sorption potential than the other two compounds (PFOS and PFOA) at both concentration levels. However, significant differences were observed among the various soil types in terms of PFBA sorption. Notably, whether applied individually or as part of a mixture (PFAS3), the sorption of PFBA was higher in soils containing PAHs (PH soil). Figure 1c and d reveal the sorption of each compound individually in the soils at two concentrations levels. With a similar pattern in both concentrations, PFOS showed the highest sorption rates followed by PFOA and, finally, PFBA showed the lowest sorption. Generally, two major observations were evident in the individual adsorption of PFAS. Firstly, the dominance of PFOS sorption across all soil types was noted, corroborating previous research that reported increased sorption with longer fluorocarbon chains (Campos Pereira et al. 2018; Milinovic et al. 2015). Secondly, a significant increase in PFAS sorption was observed in PH soil compared to CM and PT soils (Fig. 1c, d; Table 1).

The distribution coefficients (log *K*_d_) presented in Table S8 indicate that sorption increased in the order of PT < CM < PH for all three compounds, with PFBA exhibiting consistently lower sorption compared to PFOS and PFOA. The organic carbon-normalized distribution coefficients (log *K*_oc_) further show that PT soil, despite having the highest organic carbon content (45.1%), exhibited the lowest *K*_oc_ values, while PH soil (29.8% OC) had the highest PFAS sorption. This suggests that PFAS sorption is governed by additional properties beyond the content of organic carbon in soils, in agreement with previous studies (Campos Pereira et al. 2018; Milinovic et al. 2015; Zhao et al. 2014). Furthermore, the results of XPS analysis (Figure S1) qualitatively confirm the adsorption of PFAS onto the soil surfaces. While a peak around 686 eV represents native fluorinated compounds in the soils, PFAS adsorption appears as peaks around 689 eV, indicating CF and CF₃-related signals in the treated soils and the binding of fluorinated PFAS compounds to the surface. Among the soils, CM and PH exhibit stronger F1s signals, implying a relatively higher affinity for PFAS adsorption at the surface, which aligns with the findings on PFAS adsorption (Fig. 1). Meanwhile, it should be noted that the part of lower affinity of PFAS to PT soil may be attributed to the high concentration of DOM in this soil. Previous research have identified DOM as a potential competitor for PFAS, which can enhance the solubility of PFAS in the aqueous phase by decreasing solid/liquid partitioning and *K*_d_ values (Kookana et al. 2023; McCleaf et al. 2017).

The higher PFAS sorption capacity of PH soil, compared to the other two soils, could be explained from two main perspectives. Firstly, the ^13^C NMR analysis revealed differences in the quality of organic matter (OM), with lipid-to-carbohydrate ratios approximately three times higher than those in the other soils (Table 1), possibly due to the high concentration of polyaromatic hydrocarbon compounds (Table S3). This indicates that the SOM present in PH soils had a more hydrophobic nature, which has been previously reported to have a significant role in PFAS sorption (Cofield et al. 2007; Yin et al. 2008). Secondly, increased levels of extractable Al and Fe in PH soil (Table S1) may be associated with an abundance of Al/Fe oxides, potentially enhancing PFAS sorption compared to other soils (Campos-Pereira et al. 2022; Hellsing et al. 2016). While the ^13^C NMR analysis showed no notable differences in the carbon chemistry of CM and PT soils (Table 1), the higher PFAS sorption observed in CM soil compared to PT could be attributed to its greater concentrations of HNO_3_-extractable cations such as Al and Fe (Table S1) highlighting the role of electrostatic interactions in addition to hydrophobic partitioning (Askeland et al. 2020). Additionally, the considerably higher electrical conductivity (EC) in CM soil (Table S1) could suppress the double-layer thickness in soil particles reducing the possible electrostatic nature of the PFAS interaction with the soil matrix and therefore enhancing sorption onto the soil (Chen et al. 2012).

### DOM release affected by PFAS

#### Extent of DOM release

**Fig. 2:**
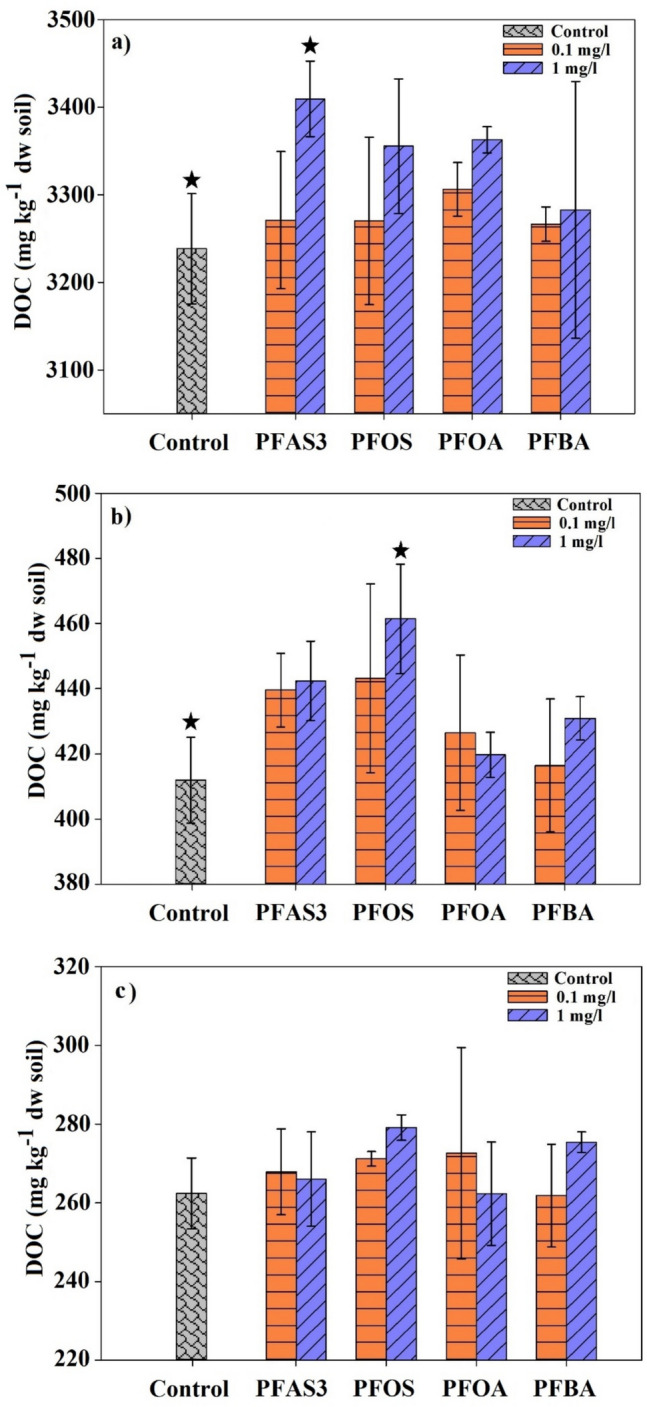
DOC release from soil affected by PFOS, PFOA, and PFBA both as a mixture and as individual compounds at two concentrations of 0.1 and 1 mg/L. **a** PT soil, **b** CM soil, and (**c**) PH soil. Error bars represent standard deviation (*n* = 3). The star above column shows significant differences compared to control by applying T-student test (*p* < 0.05)

Figure 2 shows the results of detected DOC in supernatant in the presence of PFAS both as a mixture and as individual compounds. In PT soil (Fig. 2a), PFAS3 resulted in the highest DOC release with 3409 ± 43 mg/kg, which was significantly (*p* < 0.05) higher than that of the control (3238 ± 63 mg/kg). PFOS and PFOA individually could mobilize more DOC from the soil compared to the amount mobilized in the control. Only a minimal difference in DOC release was observed between the control and the samples containing PFBA. Additionally, increased DOC release was negligible at lower PFAS concentrations but became significant at a concentration of 1 mg/L PFAS. This observation underscores the role of PFAS concentration and accumulation levels in accelerating the release of OC from the soil matrix. The impact of PFAS on the DOC release from CM soil is shown in Fig. 2b. In CM soil (Fig. 2b), it shows a slight increase in DOC release in the presence of PFAS3. However, the maximum DOC release was observed when PFOS was individually present in the solution, with a DOC concentration of 461 ± 16 mg/kg (dw soil), which was significantly (*p* < 0.05) higher than that of the control (412 ± 13 mg/kg). In contrast, both PFOA and PFBA exhibited negligible effects on DOC release in CM. It appears that in CM soil, PFOS played a dominant role in mobilizing DOC compared to the other PFAS compounds. It could be attributed to the more hydrophobic properties in PFOS due to additional C-F units in the structure of PFOS compared to PFOA and PFBA (Meng et al. 2014). Results from the PH soil were distinct from PT and CM (Fig. 2c). Although PH soil showed the highest PFAS sorption among the soils (Fig. 1a), DOC release remained negligible compared to the control, irrespective of the concentration and type of compound. The highest DOC release took place with an individual solution of PFOS, showing a release of 279 ± 3 mg/kg (dw soil), compared to 262 ± 8 mg/kg (dw soil) in the control (Fig. 2c); however, this difference was not statistically significant.

Generally, an inverse correlation between PFAS sorption in the soil and DOC was observed, meaning that as PFAS sorption increased, the concentration of DOC release from the soil decreased. This relationship seems to be driven more by the SOM composition of each soil type than by the mobilization effects associated with PFAS sorption. Notably, PT soil exhibited the lowest PFAS sorption and the highest DOC mobilization. In contrast, the soil containing PAH compounds (PH soil) exhibited the highest PFAS sorption, with around 98% of PFOS sorption, at both its low and its high concentrations, and showed the lowest DOC mobilization compared to control (Fig. 1c, d). The simultaneous release of PAHs caused by PFAS3 was negligible (Fig. S6).

The reciprocal effects of PFAS on SOM and the mobilization of OM appear to be primarily governed by hydrophobic interactions. Unlike electrostatic adsorption, which involves ionic exchange (Dragone et al. 2022; Wang et al. 2024), hydrophobic adsorption of PFAS does not necessarily mobilize SOM under all conditions and is dependent on the hydrophobic characteristics of SOM compositions. For instance, PH soil, which had more hydrophobic properties in organic matter, as shown by ^13^C NMR analysis (Table 1), showed the highest sorption of PFAS and, at the same time, lower levels of DOC release. This can be attributed to the cumulative hydrophobic sorption due to the presence of dominant compounds with hydrophobic moieties (Raber et al. 1998). A similar phenomenon was observed by Sun et al. (2009), who demonstrated that phenanthrene adsorption increased due to the cumulative and co-adsorption effects of DOM. Conversely, PT and CM soils, which exhibited lower hydrophobicity in SOM compared to PH (Table 1), showed increased PFAS mobility and higher DOM release. These results may be attributed to the surfactant behavior of PFAS, which likely stimulates the mobilization of more hydrophobic components within the SOM structure (Ahn et al. 2010; Kancharla et al. 2022).

#### DOM chemical characteristics

**Fig. 3:**
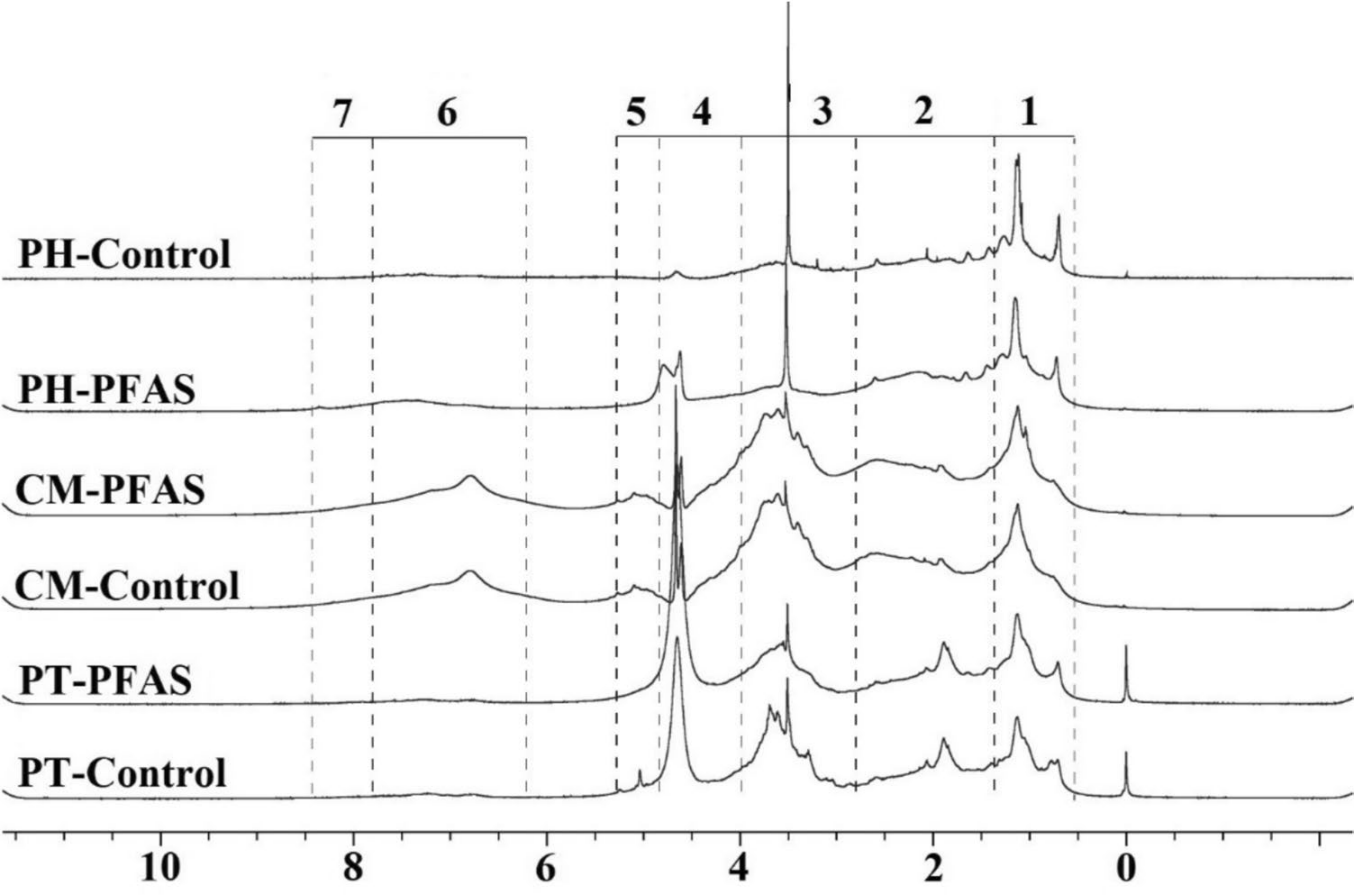
Proton nuclear magnetic resonance (^1^H NMR) spectra from the soil-extracted DOM. PFAS represents the PFAS-affected DOM and the control presents DOM extracted from control samples (PFAS-free). Region 1: aliphatic groups; region 2: N- and O-substituted aliphatic H; region 3: O-alkyl H; region 4: alpha protons of peptides; region 5: anomeric protons; region 6: aromatic/phenolic H; and Region 7: amide H in DOM-derived ^1^H NMR spectra according to the Mitchell et al. (2018). Separated spectra are presented in Figure S7

**Fig. 4:**
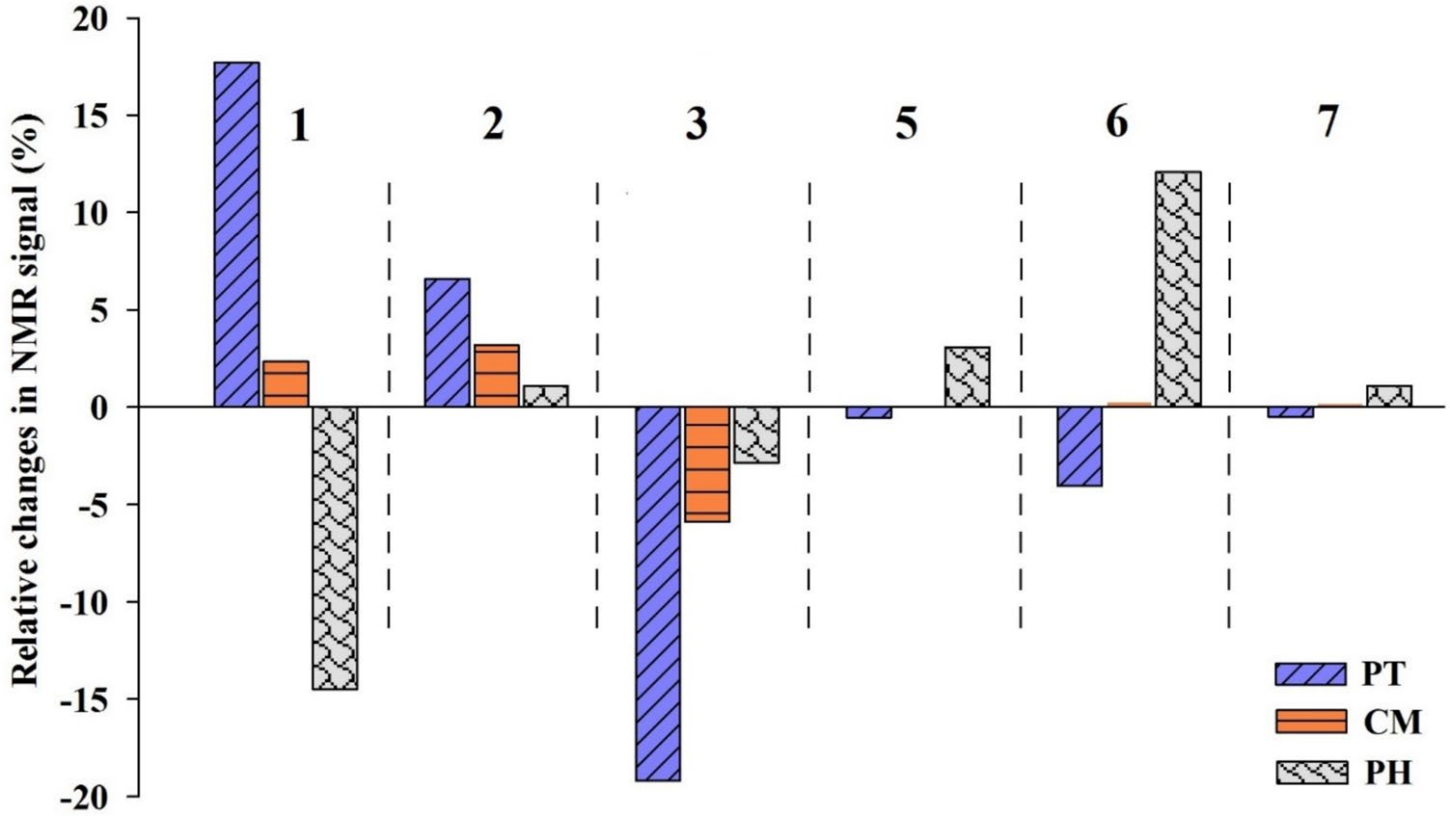
Relative changes in ^1^H NMR spectra caused PFAS (PFAS3) compared to the control (PFAS free DOM) in soil-extracted DOM. The numbers correspond to the defined H chemistry regions as explained in the caption of Fig. 3. Region 1: aliphatic groups; region 2: N- and O-substituted aliphatic H; region 3: O-alkyl H; region 4: alpha protons of peptides; region 5: anomeric protons; region 6: aromatic/phenolic H; and region 7: amide H in DOM-derived ^1^H NMR spectra according to the Mitchell et al. (2018). Due to the use of D2O in preparing DOM samples for 1H NMR analysis, region 4 is dominated by water protons and was excluded from further calculations and interpretations

To characterize and detect the possible changes in DOM chemical structure, PFAS3-affected samples and the control (PFAS-free) were characterized by ^1^H NMR. Figure 3 illustrates ^1^H NMR spectra of soil-extracted DOM in seven primary OM component categories, with the integration model describing proton chemistry associations (Mitchell et al. 2018). Figure 4 visualizes the relative changes in DOM components in ^1^H NMR spectra induced by PFAS. Positive percentage changes in each chemistry region denote the increase of the related component influenced by PFAS3, while negative percentage changes indicate a reduction of such component (Table S10). Based on known variability in run to run NMR integration observed in real operational settings, as previously reported in the literature (Stavarache et al. 2022), the present study adopted a conservative approach to minimize overinterpretation and the influence of instrumental noise. Differences in DOM region integrals smaller than 5% were considered within the method’s variability and were not interpreted as chemically meaningful. Only reproducible changes exceeding this threshold were considered indicative of PFAS-induced compositional shifts. However, for transparency, all data including those below the 5% threshold are presented in Fig. 4.

A notable alteration in the chemistry of leached DOM induced by PFAS was observed in the aliphatic constituents, i.e., CH_3_ and CH_2_ (Figs. 3 and 4, region 1). Generally, the relative change in aliphatic components was positive for PT, with an increase of + 17.7%, while for CM was + 2.35%, which is below the interpretation threshold in the present study (± 5%). This suggests that PFAS had a relatively pronounced effect on the mobilization of aliphatic compounds, such as aliphatic lipids, lignin, and polymethylene chains, such as waxes, suberin, and cutin (Mitchell et al. 2018; Simpson et al. 2007), from PT soil. The second positive percent changes observed in PT with an increase of + 6.6% attributed to alterations in N- and O-substituted aliphatic chains, specifically involving amino acids and carboxylic groups within lipids. This suggests the potential release of similar compounds in this chemical region upon PFAS presence in the system. The negative percentage changes observed in the ^1^H NMR spectrum of DOM in the PT indicate a substantial − 19.1% change in carbohydrates and proteins. The CM exhibited a pattern similar to that of PT, with a notable − 5.8% relative changes in the portion of carbohydrates and proteins in PFAS-affected DOM. The aromatic and phenolic components in PT exhibited − 4% relative changes, below the interpretation threshold in the present study. In CM, the composition of released DOM in the presence of PFAS remained relatively unchanged in other chemical regions. PFAS did not promote a change in the anomeric and amid constituents of DOM derived from PT and CM.

In PT soil, PFAS exposure enhanced the release of aliphatic constituents and reduced the release of carbohydrate components, indicating that DOM became more hydrophobic. This change can be attributed to the relatively low adsorption of PFAS onto PT soil (Fig. 1). The PFAS remaining in solution can interact with soil organic components, forming aggregates or complexes that solubilize more hydrophobic material. This process facilitates the release of hydrophobic compounds from the soil (Chen et al. 2025; Olshansky et al. 2025; Zhao et al. 2014). Previous findings support the impact of long-chain PFAS on enhancing hydrophobic interactions (Dudley 2012; Liu et al. 2020; Meng et al. 2014; Rahman et al. 2014) aligning with the present results that demonstrate the dominant role of PFOS and PFOA in increasing the hydrophobic components of DOM release from soil (Fig. 2). The results suggest that PFOS had a greater influence on DOM composition in CM soil compared to PFOA and PFBA. This is supported by its significant effect in the extent of DOC release (Fig. 2) and its highest sorption percentage (Fig. 1). The more hydrophobic nature of PFOS as compared to PFOA and PFBA, may be the most reasonable explanation supporting this result (Leung et al. 2023). In contrast, carbohydrates contribute to the polar nature of DOM and often participate in hydrophilic complex interactions (Li et al. 2023; Zhao et al. 2020), which may explain the reduced contribution of carbohydrate compounds in PFAS-affected DOM.

Although the total DOM concentrations from PH soil did not change significantly upon PFAS3 presence in the system (Fig. 2), ^1^H NMR spectra revealed a markedly changed DOM quality in the presence of PFAS (Fig. 4). As shown in Fig. 3, the NMR signal intensities of both CH_2_ and CH_3_ in the aliphatic region significantly decreased, of about − 14.4%, in PFAS-affected DOM components. Some small shifts in DOM from PH soil chemical regions, not considered significant in the present study, were observed in the O-alkyl region (− 2.8%), indicating reduced carbohydrates and proteins in DOM components, in N- and O-substituted aliphatic and amide components (~ + 2.2% in total, 1.1% each), and in anomeric constituents (+ 3%). The major alterations in DOM components of PH soil, following the addition of PFAS, were associated with the aromatic and phenolic components, exhibiting a + 12% percentage change.

The PFAS-induced changes in PH soil, namely, a decrease in the aliphatic region and an increase in aromatic signals, suggest that DOM hydrophobicity shifted toward greater aromatic/phenolic contributions rather than aliphatic groups. The high PFAS sorption in PH soil (98.4% for PFOS and 82.4% for PFOA) likely underlies this shift. The strong hydrophobicity of SOM in PH soil promotes sorption of hydrophobic compounds (Guirado et al. 2023). As a result, relatively little PFAS remained in the aqueous phase, limiting the mobilization of additional hydrophobic DOM. This is consistent with the observation that PFAS addition to PH soil did not change DOC release (Fig. 2). The observed enrichment of aromatic/phenolic components in DOM may instead result from PFAS displacing native or exogenous aromatic-rich organic matter from sorption sites into the dissolved phase, potentially linked to the soil’s PAH contamination. This is supported by the higher PAH concentrations measured in the supernatant compared to the control (Fig. S6). It could be attributed to the higher abundance and availability of PAHs in the soil, where the presence of additional hydrophobic compounds may influence their mobility within the soil matrix (Gharibzadeh et al. 2016; Li et al. 2013). As a result, PFAS appear to be ineffective at solubilizing indigenous components from SOM in strongly hydrophobic soils but could facilitate the release of xenobiotics in soil like aromatic and phenolic compounds in PAH-contaminated soil. It highlights potential risks in contaminated soils containing multiple pollutants, where hydrophobic pollutants, such as long-chain PFAS, can mobilize other contaminants within the soil profile, transferring them to groundwater and acting as a secondary source of pollution.

## Conclusion

This study investigated the reciprocal interactions between PFAS and SOM through both quantitative and qualitative assessments. The findings demonstrate that, in addition to the potential electrostatic interactions influenced by soil differences, hydrophobicity played a substantial role in PFAS sorption of within the soil matrix. Analysis of SOM composition revealed that an increase in hydrophobic components can potentially enhance PFAS sorption in soils, suggesting that OC content alone is not the primary factor determining the fate of PFAS in soils. The investigation also supported the hypothesis that PFAS has a potential to mobilize SOM. Overall, PFOS and PFOA demonstrated a significant impact on DOM release from soils, likely due to their longer carbon chains, which contributed to greater hydrophobicity. The NMR characterization of mobilized DOM indicated an increase in hydrophobic constituents, such as aliphatic compounds, in the presence of PFAS. However, the extent to which PFAS alter DOM composition varied across different soils, depending on the origin and chemistry of organic components in the soil. This study underscores the substantial risk posed by long-chain PFAS compounds in soil, as they may deplete essential SOM sources and alter its chemical composition by increasing the mobilization of organic matter. Conversely, the quality of SOM significantly influences the fate of PFAS in soil, affecting both their accumulation and their mobility in the environment. Future research should focus on modeling the interaction between SOM chemical composition and PFAS to better understand their environmental behavior on a broader scale.

## Supplementary Information

Below is the link to the electronic supplementary material.

Supplementary file 1 (DOCX 1.42 MB)

## Data Availability

The authors declare that the data supporting the findings of this study are available within the paper and its supplementary information files. Should any raw data files be needed in another format, they are available from the corresponding author upon reasonable request.
