# Supplementary material for: Reciprocal influence of per- and polyfluoroalkyl substances (PFAS) and soil organic matter on their fate in soils
Source: Environ Sci Pollut Res Int. 2025 Oct 10;32(40):23265–77. doi: 10.1007/s11356-025-37024-9 (PMC12553564; doi:10.1007/s11356-025-37024-9)
Supplement: Supplementary file 1 — (DOCX 1.42 MB) [file 11356_2025_37024_MOESM1_ESM.docx]

**Reciprocal influence of Per- and Polyfluoroalkyl Substances (PFAS) and soil organic matter on their fate in soils**

Sajjad Hazrati^1,*^, Jurate Kumpiene^1^, Tiina Leiviskä^2^, Ivan Carabante^1^

^1^Waste Science and Technology, Luleå University of Technology, Luleå, Sweden

^2^Chemical Process Engineering, University of Oulu, Oulu, Finland

* Correcsponding author.

Email addresses: [sajjad.hazrati@ltu.se](mailto:sajjad.hazrati@ltu.se), [sajjad.hazrati2@gmail.com](mailto:sajjad.hazrati2@gmail.com) (S. Hazrati)

**Contents**

S1. The H chemistry obtained from 1H NMR

S.2. Sorption coefficients (Kd and Koc)

Table S1. Soil characteristics

Table S2. PFAS analysis in soil samples.

Table S3. Summary of the analysis of Polycyclic Aromatic Hydrocarbons (PAHs) in PH soil sample.

Table S4. Surface composition (wt.%) of soil samples by XPS analysis.

Table S5. PFAS concentrations in provided initial stock solutions in two levels.

Table S6. Liquid chromatography and mass spectrometry conditions.

Table S7. Limit of quantification (LOQ) for PFAS (ng L^-1^), within ±30% of average response factor of the calibration curve.

Table S8. Sorption coefficients (Log K_d_) and organic carbon-normalized sorption coefficients (Log K_oc_) of PFOS, PFOA, and PFBA at two concentrations (0.1 and 1 mg/L) across three soil types with varying organic carbon content (PT: 45.1%, CM: 6.69%, PH: 29.81%).

Table S9. Dissolved organic carbon chemistry based on peak integrals from ^1^H NMR spectra of three different soils (Mitchell et al., 2018). The region related to the alpha protons of peptides was excluded due to the dominance of water molecules involved in sample preparation processes.

Table S10. Relative changes in ^1^H NMR spectra caused PFAS (PFAS3). Spectra obtained from the soil extracted DOM from three soils.

Figure S1. XPS F1 spectra showing fluorine binding states and surface composition in control soils and PFAS-amended soil. The peak around 686 eV corresponds to native fluorinated compounds in control samples (possibly metal fluorides), while the peak around 689 eV indicates fluorinated compounds related to C-F bonding in PFAS (Ren et al., 2023).

Figure S2. Adsorption kinetics of PFOS onto three soils over time. The figure shows the change in PFOS adsorption (Q_t_) as a function of contact time.

Figure S3. pH changes in soil leachate affected by PFAS compared to the control (PFAS-free). A) PFAS3 (mixture), b) PFOS, c) PFOA, and d) PFBA. Equilibrium time is 24 h with liquid to soil ratio of 40 ml g^-1^ dw.

Figure S4. Spectra of solid-state ^13^C Cross-Polarization Magic Angle Spinning Nuclear Magnetic Resonance (^13^C CP-MAS NMR) for soils.

Figure S5. Soil organic carbon chemistry obtained from ^13^C CP-MAS NMR analysis, based on integration model (Baldock et al., 2004).

Figure S6. PAH16 leaching from PH soil in control (PFAS-free) and PFAS3 (mixture) in two levels of concentration. Error bars present Standard deviation (n=2), except for 0.1 mg L^-1^ which was measured in a single sample.

Figure S7. Individual spectra of soil-derived DOM obtained from ^1^H NMR analysis.

**S1. The H chemistry obtained from 1H NMR**

The figure 3 presents Proton Nuclear Magnetic Resonance (1H NMR) spectra from the soil extracted DOM and figure 4 demonstrates the relative changes in H chemistry of DOM in 1H NMR spectra caused by PFAS. Hereby the chemistry of different regions on ^1^H NMR was discussed in detail. The signals originated from the aliphatic region, involving two main proton-related chemical groups. The signal at approximately 0.85 ppm corresponds to aliphatic lipids, lignin, and proteins associated with CH_3_ protons. Protons related to CH_2_ in DOM, indicating polymethylene chains such as cutin, lipids, waxes, and suberin, resonate at around 1.25 ppm (Mitchell et al., 2018; Simpson et al., 2007). The signals between 1.3 and 2.9 ppm are attributed to N- and O-substituted aliphatic resonances caused by protons in CH2 groups proximate to polar functional groups. This includes β or γ carboxylic acid groups in lipids or in amino acid side chains. This spectral region also includes short-chain carboxylic acids produced through microbial activity during organic matter degradation (Kelleher & Simpson, 2006a; Pisani et al., 2015). The O-alkyl region (Region 3) is defined by protons linked to carbohydrates, with minor contributions from protons in proteins and methoxy groups of lignin. Anomeric proton signals associated with carbohydrates are observable in the region between 4.8 – 5.2 ppm (Kelleher & Simpson, 2006b). The protons in lignin derived compounds and aromatic amino acids like tyrosine and phenylalanine originate aromatic and phenolic signals in 1H NMR spectra (Pautler et al., 2013).

**S.2. Sorption coefficients (K_d_ and K_oc_)**

The distribution coefficient (Kd) was determined for PFOS, PFOA, and PFBA in the three soil types. Kd expresses the ratio of a compound’s concentration sorbed to soil to its concentration in the aqueous phase, and is calculated using the following equation:

$$K_{d}= \frac{C_{s}}{C_{w}}$$

Where, 𝐶𝑠 is the concentration of PFAS in the soil (mg/kg) and Cw is the concentration of PFAS in the aqueous phase (mg/L).

The organic carbon-normalized distribution coefficient (Koc) was then calculated to account for the influence of soil organic matter, using the equation:

$$K_{oc}= \frac{K_{d}}{f_{oc}}$$

Where, f_oc_ is the mass fraction of organic carbon in the soil.

**Table S1.** Soil characteristics.

| Soil | Unit | PT | CM | PH |
| --- | --- | --- | --- | --- |
| pH (H_2_O)^a^ | - | 4.25 | 6.05 | 4.52 |
| EC^b^ | µs cm^-1^ | 164 | 8020 | 1465 |
| Organic carbon^c^ | % dw | 45.1 | 6.69 | 29.87 |
| Dissolved organic carbon^d^ | mg kg^-1^ | 3238.2 | 412 | 262.3 |
| 0.1 M BaCl_2_ extractable cations^e^ | mmol kg^-1^ dw |  |  |  |
| Al |  | 3.98 | 0.73 | 26 |
| Fe |  | 1.23 | 0.56 | 2.33 |
| Ca |  | 64.2 | 38 | 75.4 |
| Mg |  | 13.7 | 7.06 | 12 |
| K |  | 5.16 | 13.8 | 6.9 |
| 0.1 M HNO_3_ extractable cations^f^ | mmol kg^-1^ dw |  |  |  |
| Al |  | 8.63 | 44.5 | 177 |
| Fe |  | 5.41 | 50.5 | 58.7 |
| Ca |  | 109 | 137 | 166 |
| Mg |  | 21.2 | 12 | 17.5 |
| K |  | 9.07 | 16.2 | 6.9 |

a Liquid to soil ratio of 40 ml g^-1^ dw.

b Electrical conductivity measured in leachate extracted from saturated soil.

c Soil total organic carbon measured by solid-state total organic carbon (TOC) analyzer

d Extracted from soil with a liquid to soil ratio of 40 ml g-1 dw in 24 h.

e 2.5 g dw was suspended in 100 mL 0.1 M BaCl_2_, and equilibrated for 4 h.

f 1.0 g dw was suspended in 30 mL 0.1 M HNO_3_, and equilibrated for 16 h.

**Table S2.** PFAS analysis in soil samples.

| Acronym | Compounds | PT  (µg/kg dw) | CM  (µg/kg dw) | PH  (µg/kg dw) |
| --- | --- | --- | --- | --- |
| PFBA | Perfluorobutanoic acid | 0.75 | 0.12 | <0.22 |
| PFPeA | perfluoropentanoic acid | 3.8 | 0.14 | <0.066 |
| PFHxA | Perfluorohexanoic acid | 1.5 | 0.55 | <0.066 |
| PFHpA | perfluoroheptanoic acid | <0.12 | <0.030 | <0.066 |
| PFOA | Perfluorooctanoic acid | <0.40 | 0.28 | <0.066 |
| PFNA | Perfluorononanoic acid | <0.12 | <0.030 | <0.066 |
| PFDA | Perfluorodecanoic acid | <0.40 | 0.13 | <0.22 |
| PFBS | Perfluorobutanesulfonic acid | 0.93 | 0.12 | <0.066 |
| PFHxS | Perfluorohexanesulfonic acid | <0.12 | <0.030 | <0.066 |
| PFOS | Perfluorooctanesulfonic acid | <0.40 | 0.51 | <0.066 |
| 6:2 FTS | 6:2-fluorotelomersulfonic acid | <0.12 | 0.065 | <0.066 |
| ∑PFAS 11 |  | 7.8 | 2 | <0.52 |
| ∑PFAS 4 |  | <0.52 | 0.82 | <0.14 |

**Table S3.** Summary of the analysis of Polycyclic Aromatic Hydrocarbons (PAHs) in PH soil sample.

| **Compound** | **Results** | **STD** | **Units** |
| --- | --- | --- | --- |
| Naphthalene | 22.4 | ± 6.8 | mg kg^-1^ dw |
| Acenaphthylene | 12.1 | ± 3.7 | mg kg^-1^ dw |
| Acenaphthene | 111 | ± 33.7 | mg kg^-1^ dw |
| Fluorene | 243 | ± 74 | mg kg^-1^ dw |
| Fenantren | 684 | ± 208 | mg kg^-1^ dw |
| Anthracene | 619 | ± 188 | mg kg^-1^ dw |
| Fluoranten | 3700 | ± 1120 | mg kg^-1^ dw |
| Pyrene | 1980 | ± 603 | mg kg^-1^ dw |
| Benz[a]anthracene | 522 | ± 159 | mg kg^-1^ dw |
| Chrysene | 477 | ± 145 | mg kg^-1^ dw |
| Benzo[b]fluoranthene | 185 | ± 56.2 | mg kg^-1^ dw |
| Benzo[k]fluoranthene | 61.8 | ± 18.8 | mg kg^-1^ dw |
| Benzo[a]pyrene | 86 | ± 26.2 | mg kg^-1^ dw |
| Dibenz[a,h]anthracene | 6.73 | ± 2.07 | mg kg^-1^ dw |
| Benzo[g,h,i]perylene | 13.1 | ± 4.01 | mg kg^-1^ dw |
| Indeno[1,2,3-cd]pyrene | 16.3 | ± 4.97 | mg kg^-1^ dw |
| Sum PAH 16 | 8740 | ± 1324 | mg kg^-1^ dw |

**Table S4.** Surface composition (wt.%) of soil samples by XPS analysis.

| Element | PT | PT-PFAS | CM | CM-PFAS | PH | PH-PFAS |
| --- | --- | --- | --- | --- | --- | --- |
| O | 34.3 | 37.8 | 40.7 | 36.3 | 35.4 | 35.6 |
| C | 63.3 | 55.9 | 36.8 | 42.2 | 46.2 | 45.0 |
| Si | - | 3.2 | 9.2 | 4.9 | 10.9 | 10.7 |
| N | 2.4 | 2.6 | 4.4 | 6.0 | 2.8 | 2.5 |
| Al | - | - | 3.6 | 2.4 | 2.5 | 3.4 |
| Fe | - | - | 2.8 | 2.9 | 2.2 | 2.5 |
| P | - | - | 1.3 | 2.5 | - | - |
| Ca | - | 0.5 | 0.9 | 1.8 | - | 0.3 |
| K | - | - | 0.3 | - | - | - |
| F | - | -* | -* | 1.2 | -* | - * |

* The presence of fluorine was proved from high resolution F1s spectrum.

**Table S5.** PFAS concentrations in provided initial stock solutions in two levels.

| Compound | Concentration in 0.1 mg L^-1^ stock solution | Concentration in 1 mg L^-1^ stock solution |
| --- | --- | --- |
| PFAS mixture stock (PFAS3) | (µg L^-1^) | (µg L^-1^) |
| Total | 106.77 | 1190.45 |
| PFOS | 29.45 | 355.91 |
| PFOA | 33.14 | 343.64 |
| PFBA | 44.18 | 490.91 |
| Individual compound stock |  |  |
| PFOS | -^*^ | 966.49 |
| PFOA | -^*^ | 921.30 |
| PFBA | -^*^ | 1263.67 |

* The 1 mg L^-1^ stock solution was used to prepare 0.1 mg L^-1^ PFAS samples.

Stock solutions were prepared in Milli-Q water without the addition of methanol, to avoid potential interference of methanol in the adsorption and DOC experiments.

**Table S6**. Liquid chromatography and mass spectrometry conditions.

| **Parameter** | **Value** | |
| --- | --- | --- |
| LC | ACQUITY Premier System with Multi-Dimensional Technology | |
| Analytical column | ACQUITY Premier CSH Phenyl-Hexyl 2.1 x 100 mm, 1.7 µm | |
| Delayed column | ACQUITY Premier CSH Phenyl-Hexyl  2.1 x 50 mm, 1.7 µm | |
| Column temperature | 35 °C | |
| Injection volume | 10 µL | |
| Mobile phase | A) 2 mM Ammonium acetate in 95:5% Milli-Q water:MeOH B) 2 mM Ammonium acetate in 100% MeOH | |
| Flow rate | 0.3 mL/min | |
| Gradient | Time (min) Flow (ml/min) %A %B | |
|  | Initial 0.3 100 0  1 0.3 80 20 | |
|  | 6 0.3 55 45 | |
|  | 13 0.3 20 80 | |
|  | 14 0.4 5 95 | |
|  | 17 0.4 5 95  18 0.3 100 0  22 0.3 100 0 | |
| Stop time | 17 minutes | |
| Post time | 6 minutes | |
| MS Instrument conditions (UniSpray+) | |  |
| **Parameter** | **Value** | |
| MS | Xevo TQ-XS Triple Quadrupole Mass Spectrometer | |
| **Voltages:**  Impactor Voltage (kV)  Cone (V) | 1  30 | |
| **Tempratures:**  Desolvation Temprature (°C) | 600 | |
| **Gas Flow:**  Desolvation (L/Hr)  Cone (L/Hr)  Nebuliser (Bar) | 1000  150  7 | |
| **Analyser:**  LM Resolution 1  HM Resolution 1  Ion Energy 1  LM Resolution 2  HM Resolution 2  Ion Energy 2  Collision Gas Flow (ml/min)  Collision) | 2.67  15.08  -0.3  2.71  14.94  0.8  0.17  4 | |

**Table S7.** Limit of quantification (LOQ) for PFAS (ng L^-1^), within ±30% of average response factor of the calibration curve.

| Compounds | Acronym | Formula | LOQ (ng/L)* |
| --- | --- | --- | --- |
| Perfluorooctanesulfonic acid | PFOS | CF_3_(CF_2_)_7_SO_3_Na | 49.0 |
| Perfluorooctanoic acid | PFOA | CF_3_(CF_2_)_6_COOH | 58.7 |
| Perfluorobutanoic acid | PFBA | CF_3_(CF_2_)_2_COOH | 59.7 |

*LOQ was calculated using the formula: LOQ = (10 × σ) / S, where σ represents the standard deviation of the responses of the standards, and S is the slope of the calibration curve.

**Table S8.** Sorption coefficients (Log K_d_) and organic carbon-normalized sorption coefficients (Log K_oc_) of PFOS, PFOA, and PFBA at two concentrations (0.1 and 1 mg/L) across three soil types with varying organic carbon content (PT: 45.1%, CM: 6.69%, PH: 29.81%).

|  | **Log Kd** | | | | | |  | **Log Koc** | | | | | |
| --- | --- | --- | --- | --- | --- | --- | --- | --- | --- | --- | --- | --- | --- |
|  | PFOS | | PFOA | | PFBA | |  | PFOS | | PFOA | | PFBA | |
|  | 0.1 mg/L | 1 mg/L | 0.1 mg/L | 1 mg/L | 0.1 mg/L | 1 mg/L |  | 0.1 mg/L | 1 mg/L | 0.1 mg/L | 1 mg/L | 0.1 mg/L | 1 mg/L |
| **PT** | 1.832 | 1.802 | 0.940 | 0.862 | 0.485 | 0.465 |  | 2.178 | 2.148 | 1.285 | 1.208 | 0.831 | 0.811 |
| **CM** | 2.329 | 2.269 | 1.055 | 1.125 | 0.689 | 0.736 |  | 3.503 | 3.443 | 2.229 | 2.299 | 1.864 | 1.911 |
| **PH** | 3.410 | 3.141 | 2.274 | 2.008 | 1.091 | 1.044 |  | 3.935 | 3.666 | 2.799 | 2.533 | 1.615 | 1.569 |

**Table S9**. Dissolved organic carbon chemistry based on peak integrals from ^1^H NMR spectra of three different soils (Mitchell et al., 2018). The region related to the alpha protons of peptides was excluded due to the dominance of water molecules involved in sample preparation processes.

| Integrated H domains | Chemical shift region (ppm) | PT-  Control | PT-PFAS affected | CM-Control | CM-PFAS affected | PH-Control | PH-PFAS affected |
| --- | --- | --- | --- | --- | --- | --- | --- |
| Aliphatic H (%) | 0.6-1.3 | 13.8 | 31.6 | 24.2 | 26.5 | 35.3 | 20.8 |
| N- and Osubstituted aliphatic H (%) | 1.3-2.9 | 30.5 | 37.1 | 32.9 | 36.0 | 39.5 | 40.6 |
| O-alkyl H (%) | 2.9-4.1 | 34.6 | 15.4 | 32.4 | 26.5 | 16.6 | 13.8 |
| Peptides α H | 4.1-4.8 | - | - | - | - | - | - |
| Anomeric H (%) | 4.8-5.2 | 2.4 | 1.8 | 2.1 | 2.1 | 0.9 | 4.0 |
| Aromatic/Phenolic H (%) | 6.2-7.8 | 15.6 | 11.6 | 6.8 | 7.0 | 6.6 | 18.6 |
| Amide H (%) | 7.8-8.4 | 3.0 | 2.5 | 1.7 | 1.9 | 1.1 | 2.2 |

**Table S10.** Relative changes in ^1^H NMR spectra caused PFAS (PFAS3). Spectra obtained from the soil extracted DOM from three soils.

| Integrated H domains | PT (%) | CM (%) | PH (%) |
| --- | --- | --- | --- |
| Aliphatic H | 17.70 | 2.35 | -14.49 |
| N- and Osubstituted aliphatic H | 6.58 | 3.18 | 1.08 |
| O-alkyl H | -19.19 | -5.88 | -2.83 |
| Anomeric H | -0.55 | -0.04 | 3.07 |
| Aromatic/Phenolic H | -4.03 | 0.25 | 12.07 |
| Amide H | -0.50 | 0.13 | 1.09 |


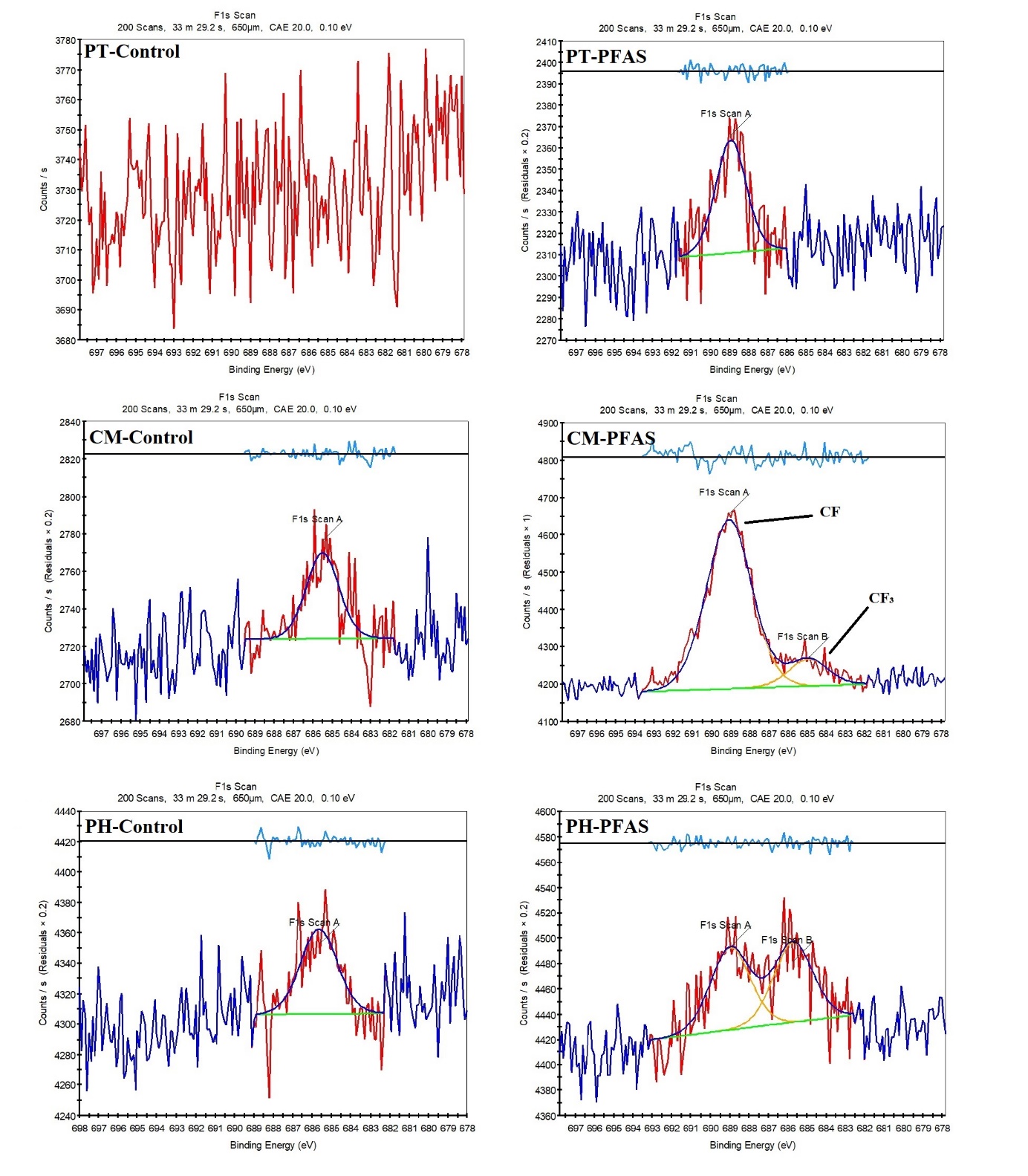


Figure S1. XPS F1 spectra showing fluorine binding states and surface composition in control soils and PFAS-amended soil. The peak around 686 eV corresponds to native fluorinated compounds in control samples (possibly metal fluorides), while the peak around 689 eV indicates fluorinated compounds related to C-F bonding in PFAS (Ren et al., 2023).


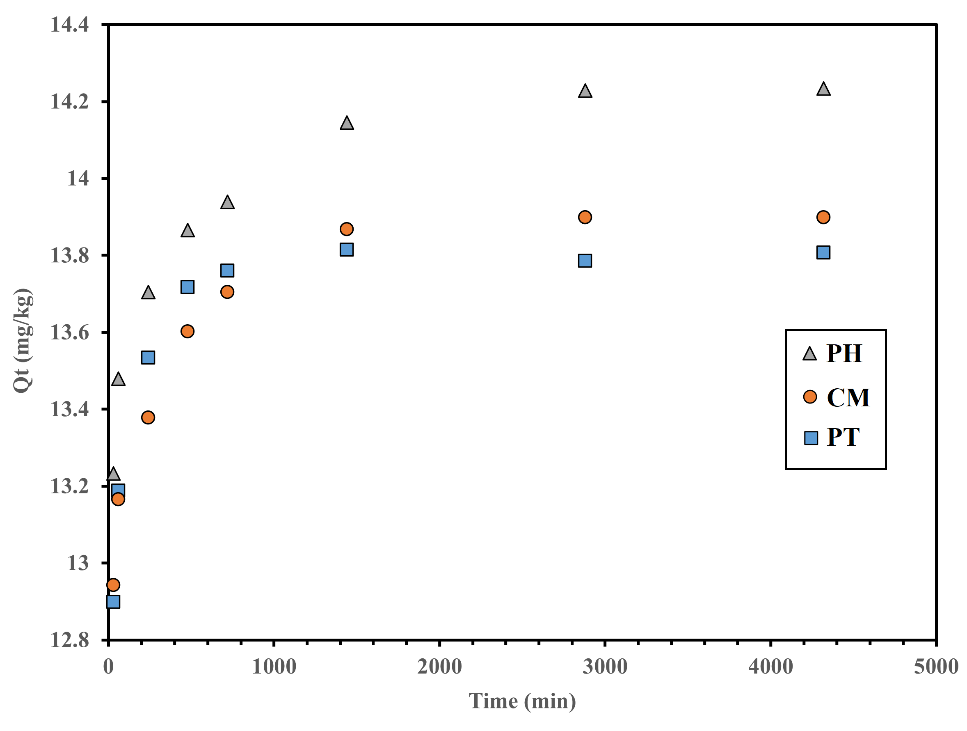


Figure S2. Adsorption kinetics of PFOS onto three soils over time. The figure shows the change in PFOS adsorption (Qt) as a function of contact time.


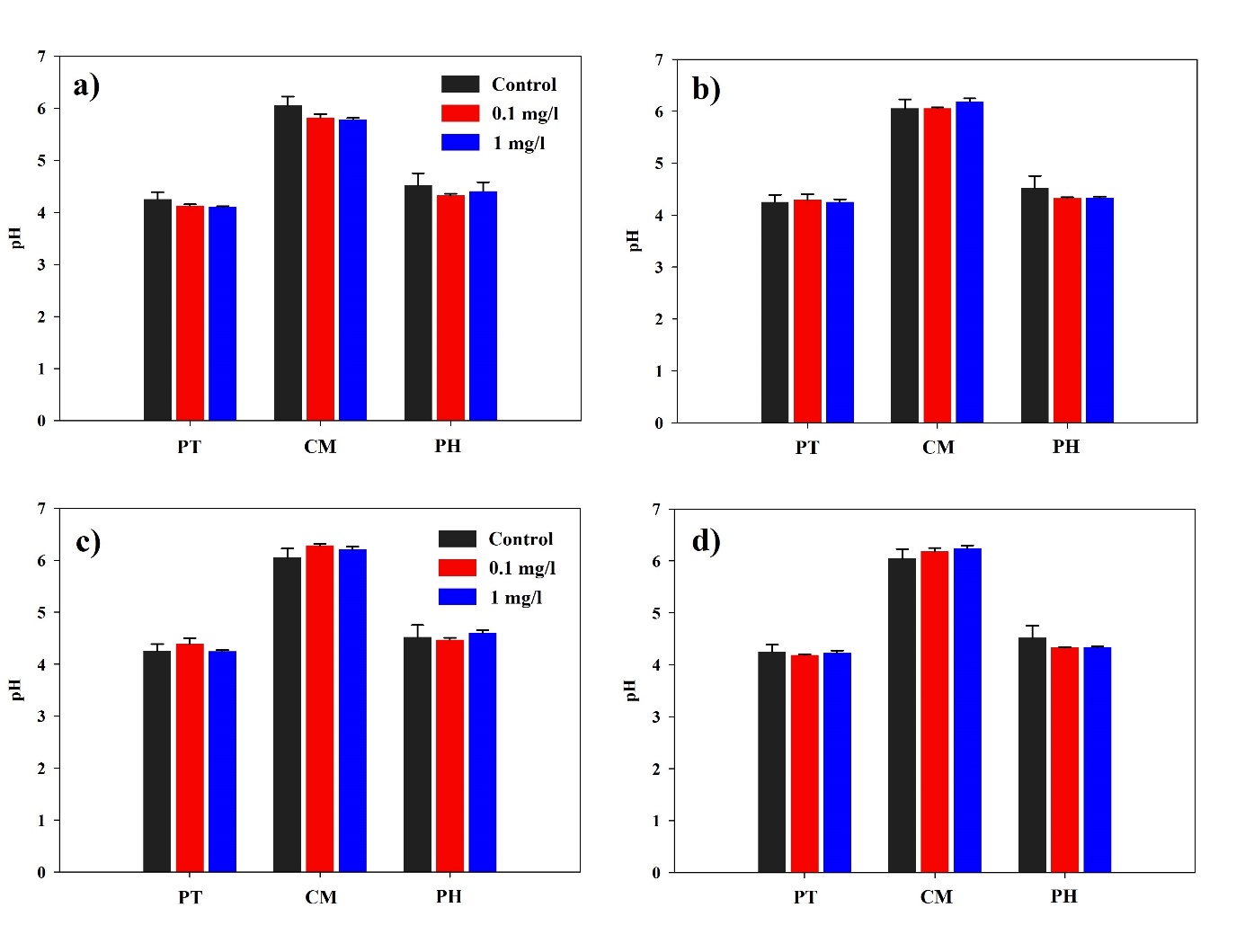


Figure S3. pH changes in soil leachate affected by PFAS compared to the control (PFAS-free). A) PFAS3 (mixture), b) PFOS, c) PFOA, and d) PFBA. Equilibrium time is 24 h with liquid to soil ratio of 40 ml g^-1^ dw.


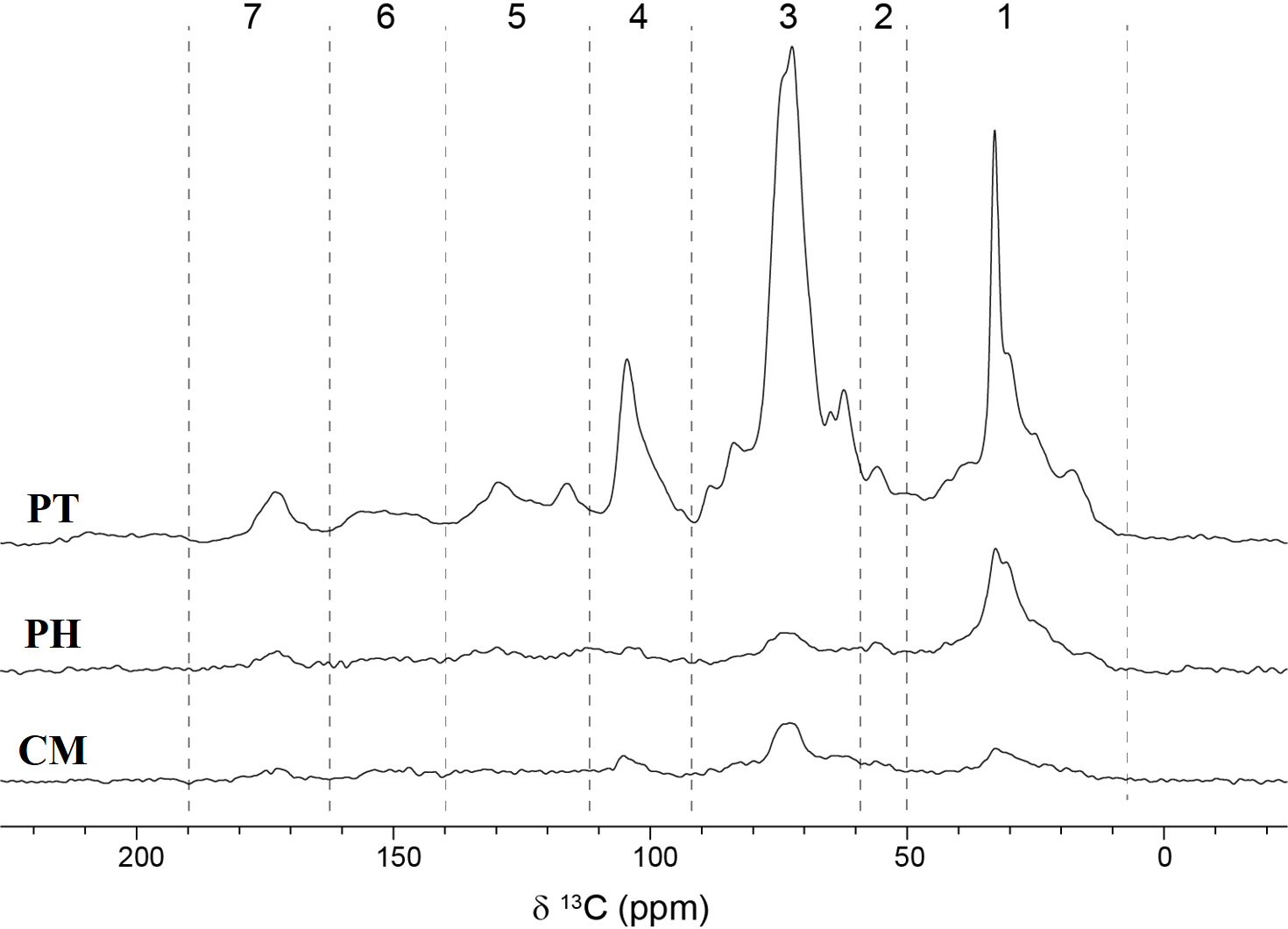


Figure S4. Spectra of solid-state ^13^C Cross-Polarization Magic Angle Spinning Nuclear Magnetic Resonance (^13^C CP-MAS NMR) for soils.


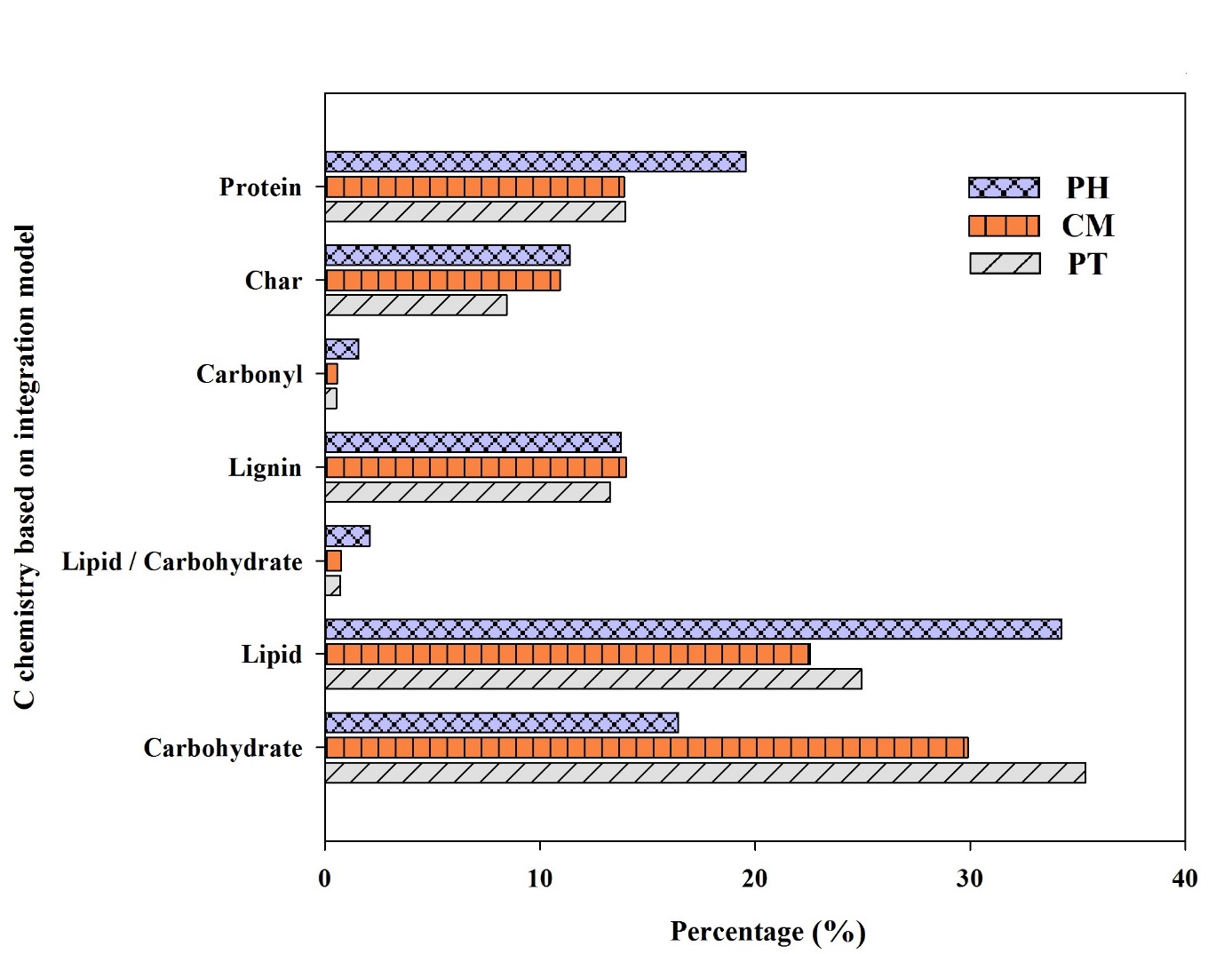


Figure S5. Soil organic carbon chemistry obtained from ^13^C CP-MAS NMR analysis, based on integration model (Baldock et al., 2004).


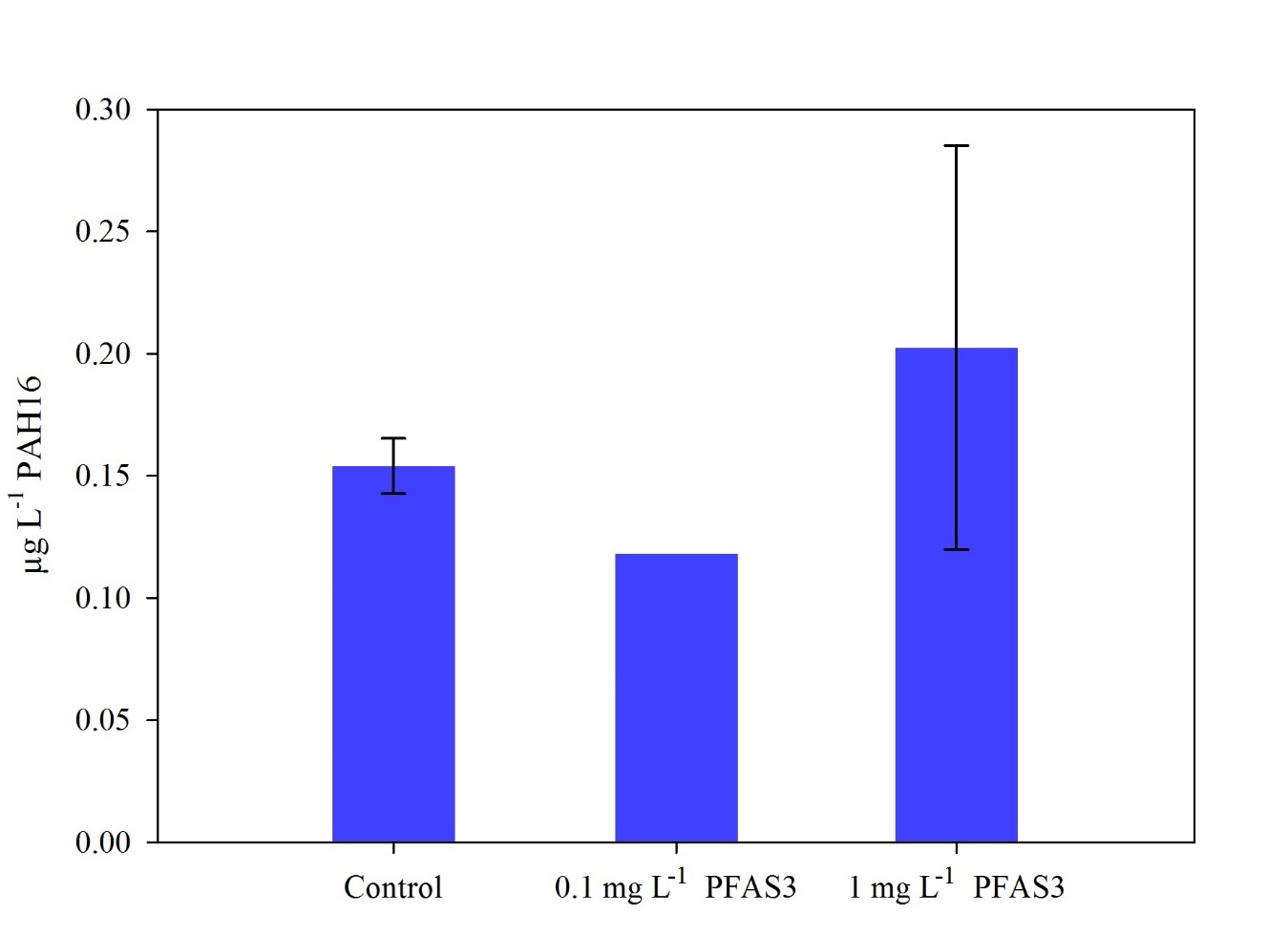


Figure S6. PAH16 leaching from PH soil in control (PFAS-free) and PFAS3 (mixture) in two levels of concentration. Error bars present Standard deviation (n=2), except for 0.1 mg L^-1^ which was measured in a single sample.


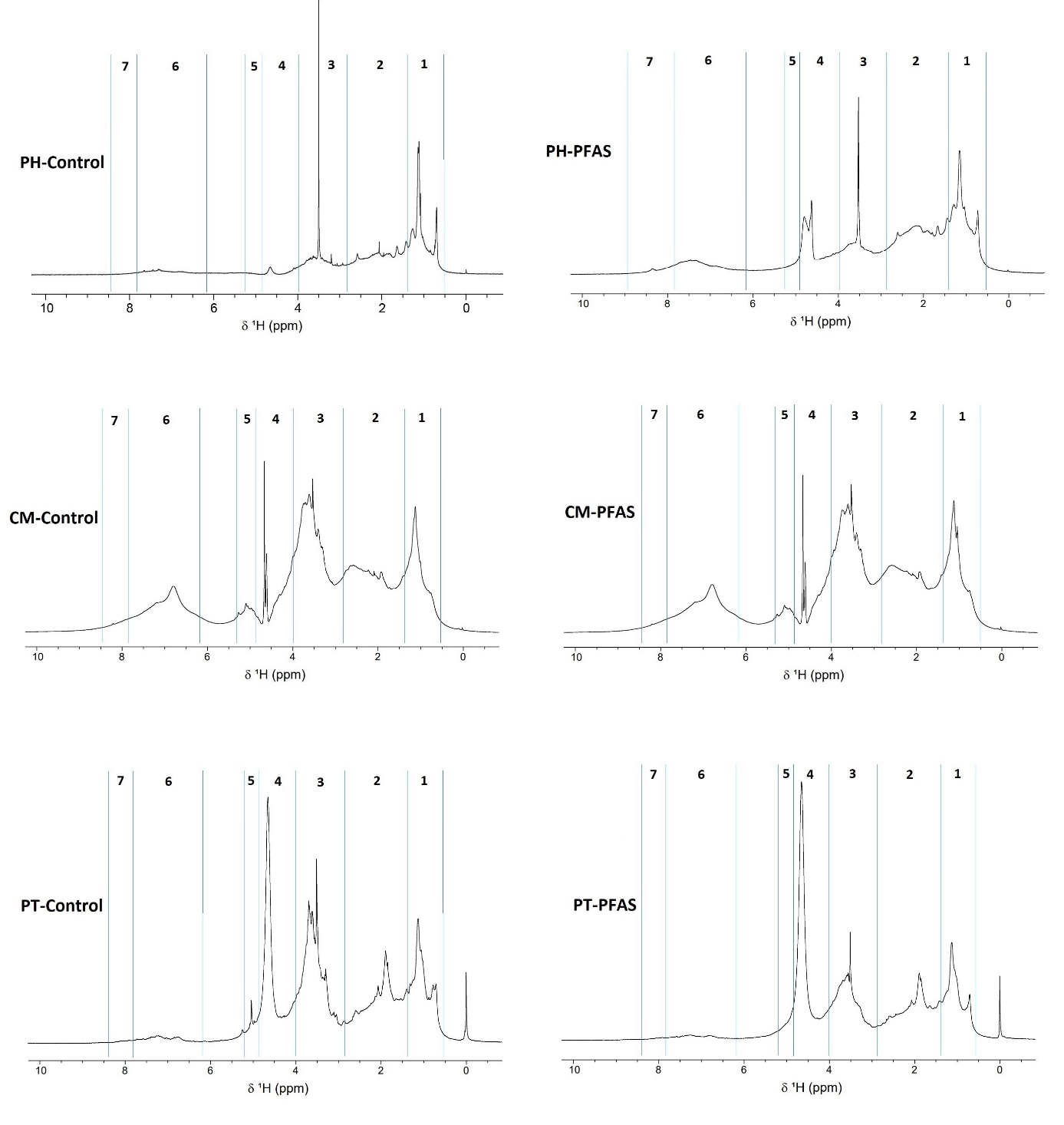


Figure S7. Individual spectra of soil-derived DOM obtained from ^1^H NMR analysis.

**References**

Baldock, J. A., Masiello, C. A., Gélinas, Y., & Hedges, J. I. (2004). Cycling and composition of organic matter in terrestrial and marine ecosystems. *Marine Chemistry*, *92*(1), 39–64. https://doi.org/https://doi.org/10.1016/j.marchem.2004.06.016

Kelleher, Brian. P., & Simpson, Andre. J. (2006a). Humic Substances in Soils:  Are They Really Chemically Distinct? *Environmental Science & Technology*, *40*(15), 4605–4611. https://doi.org/10.1021/es0608085

Kelleher, Brian. P., & Simpson, Andre. J. (2006b). Humic Substances in Soils:  Are They Really Chemically Distinct? *Environmental Science & Technology*, *40*(15), 4605–4611. https://doi.org/10.1021/es0608085

Mitchell, P. J., Simpson, A. J., Soong, R., & Simpson, M. J. (2018). Nuclear Magnetic Resonance Analysis of Changes in Dissolved Organic Matter Composition with Successive Layering on Clay Mineral Surfaces. *Soil Systems*, *2*(1). https://doi.org/10.3390/soils2010008

Pautler, B. G., Dubnick, A., Sharp, M. J., Simpson, A. J., & Simpson, M. J. (2013). Comparison of cryoconite organic matter composition from Arctic and Antarctic glaciers at the molecular-level. *Geochimica et Cosmochimica Acta*, *104*, 1–18. https://doi.org/https://doi.org/10.1016/j.gca.2012.11.029

Pisani, O., Frey, S. D., Simpson, A. J., & Simpson, M. J. (2015). Soil warming and nitrogen deposition alter soil organic matter composition at the molecular-level. *Biogeochemistry*, *123*(3), 391–409. https://doi.org/10.1007/s10533-015-0073-8

Ren, Z., Bergmann, U., Uwayezu, J. N., Carabante, I., Kumpiene, J., Lejon, T., & Leiviskä, T. (2023). Combination of adsorption/desorption and photocatalytic reduction processes for PFOA removal from water by using an aminated biosorbent and a UV/sulfite system. *Environmental Research*, *228*, 115930. https://doi.org/https://doi.org/10.1016/j.envres.2023.115930

Simpson, A. J., Song, G., Smith, E., Lam, B., Novotny, E. H., & Hayes, M. H. B. (2007). Unraveling the Structural Components of Soil Humin by Use of Solution-State Nuclear Magnetic Resonance Spectroscopy. *Environmental Science & Technology*, *41*(3), 876–883. https://doi.org/10.1021/es061576c
